# Prospects for coherent X-ray diffraction imaging at fourth-generation synchrotron sources

**DOI:** 10.1107/S2052252525001526

**Published:** 2025-03-13

**Authors:** Yuriy Chushkin, Federico Zontone

**Affiliations:** a ESRF – The European Synchrotron, 71 avenue des Martyrs, 38000Grenoble, France; UCL, United Kingdom

**Keywords:** coherent X-ray diffraction imaging, CXDI, materials science, nanoscience, X-ray microscopy, synchrotron radiation

## Abstract

Coherent X-ray diffraction imaging is a lens-less microscopy technique that emerged with the advent of third-generation synchrotrons, modern detectors and computers. It can image isolated micrometre-sized objects with a spatial resolution of a few nanometres. The method is based on the inversion of the speckle pattern in the far field produced by the scattering from the object under coherent illumination. The retrieval of the missing phase is performed using an iterative algorithm that numerically phases the amplitudes from the intensities of speckles measured with sufficient oversampling. Two- and three-dimensional imaging is obtained by simple inverse Fourier transform. This lens-less imaging technique has been applied to various specimens for their structural characterization on the nanoscale. Here, we review the theoretical and experimental elements of the technique, its achievements, and its limitations at third-generation synchrotrons. We also discuss the new opportunities offered by modern fourth-generation synchrotrons and outline the developments necessary to maximize the potential of the technique.

## Abstract 2

A review of plane-wave coherent X-ray diffraction imaging in small-angle X-ray scattering geometry is presented, together with a discussion of the new opportunities offered by fourth-generation synchrotron sources.

## 1. Introduction

There has been growing interest in using X-rays as a probe for high-resolution imaging. According to the Abbe resolution criterion, the short wavelength of X-rays enables high resolving power, allowing them to penetrate the internal structure of thick optically opaque specimens. Thanks to these properties, X-ray imaging is used intensively in many fields of applications and is being constantly improved and developed (Baruchel *et al.*, 2008 ▸; Jacobsen, 2019 ▸). In contrast to visible light microscopy, the use of lenses for image formation in the X-ray regime is limited by the challenges of manufacturing high-quality lenses and their resulting low efficiency (Chao *et al.*, 2005 ▸). The advent of powerful X-ray sources such as synchrotrons and free-electron lasers paved the way for the emergence of lens-less imaging techniques that use X-ray beams with a high degree of coherence. In particular, coherent X-ray diffraction imaging (CXDI), demonstrated in 1999 ▸ (Miao *et al.*, 1999 ▸), enabled innovative high-resolution X-ray imaging modalities capable of achieving nanometre-scale resolution without the need for lenses. With this method, an image of a specimen is recovered using numerical algorithms applied to its diffraction pattern.

Modern detectors, the development of phase-retrieval algorithms and the boosting of computational power laid the foundations for developing new coherent X-ray imaging techniques (Chapman & Nugent, 2010 ▸; Cloetens *et al.*, 1999 ▸). This led to the implementation of two classes of X-ray microscopy. The first recovers an image from diffraction patterns recorded in the near-field (Fresnel diffraction) regime, with notable techniques including in-line holography (Cloetens *et al.*, 1999 ▸; Mokso *et al.*, 2007 ▸; Soltau *et al.*, 2021 ▸; Kalbfleisch *et al.*, 2022 ▸), speckle tracking imaging (Bérujon *et al.*, 2012 ▸) and near-field ptychography (Stockmar *et al.*, 2013 ▸). The resolution of the obtained images is given by the detector pixel size and/or source size. The second class of techniques uses far-field diffraction patterns (Fraunhofer diffraction) to recover an image at the resolution given by the largest scattering angle where the diffraction signal is observed. Among the implemented methods are CXDI in forward-scattering geometry for microscopy of isolated non-crystalline specimens (Miao *et al.*, 1999 ▸; Chapman *et al.*, 2006 ▸), CXDI in Bragg geometry for imaging strain fields in nanocrystals (Pfeifer *et al.*, 2006 ▸; Robinson & Harder, 2009 ▸; Sun & Singer, 2024 ▸) and their shapes (Robinson *et al.*, 2001 ▸), Fourier transform holography (Stroke & Falconer, 1964 ▸; McNulty *et al.*, 1992 ▸; Eisebitt *et al.*, 2004 ▸) and scanning coherent imaging via far-field ptychography to visualize extended specimens (Rodenburg *et al.*, 2007 ▸; Thibault *et al.*, 2008 ▸; Dierolf *et al.*, 2010 ▸; Pfeiffer, 2018 ▸). Owing to the robustness of the ptychographic reconstruction algorithm, ptychography became a leading technique for 3D quantitative high-resolution imaging (Holler *et al.*, 2017 ▸; Aidukas *et al.*, 2024 ▸).

Clearly, all the above-mentioned imaging techniques can gain a lot from the upgrades of synchrotron sources that have been accomplished recently or that are currently underway worldwide. In this paper, we focus on CXDI in forward-scattering geometry. The development of CXDI has been driven by the prospect of single-particle imaging of biomolecules at X-ray free-electron laser (XFEL) facilities (Neutze *et al.*, 2000 ▸). At synchrotrons, CXDI has found its utility for 3D imaging of isolated microscopic specimens, and its capabilities can be further enhanced at fourth-generation synchrotron sources.

## 2. Numerical lens

**Figure 1:**
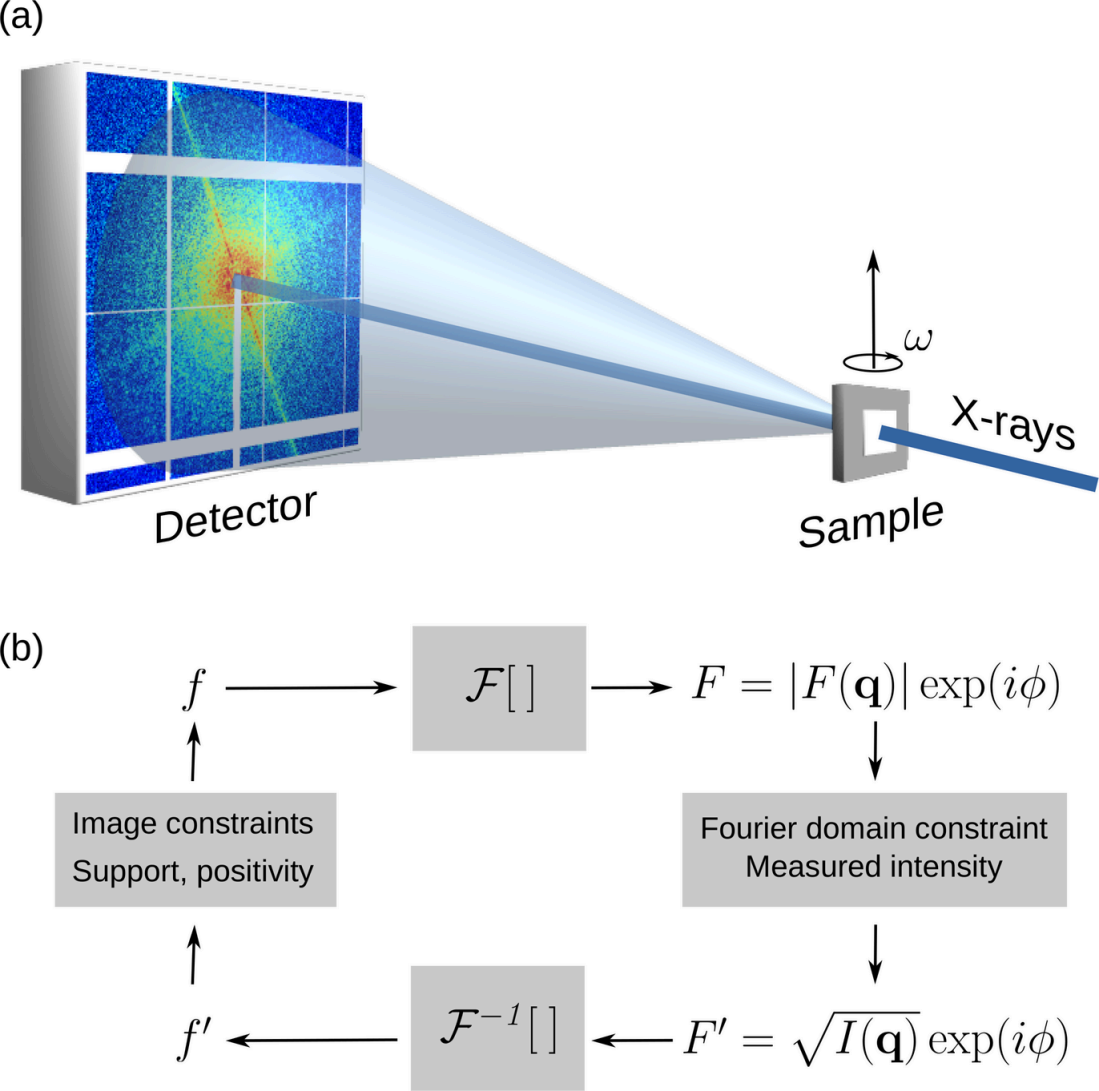
(*a*) Experimental geometry of CXDI. (*b*) Iterative phase retrieval algorithm scheme – numerical lens.

Classical microscopy schemes use lenses for magnified image formation. The manufacture of X-ray lenses is technically challenging and even the best quality lenses still can suffer from low efficiency (Chao *et al.*, 2005 ▸; Bajt *et al.*, 2018 ▸). CXDI operates without objective lenses. Fig. 1 ▸(*a*) shows the standard experimental geometry for plane-wave CXDI. An isolated specimen is placed on a thin Si_3_N_4_ membrane (transparent to X-rays) and can be rotated about the vertical axis for tomographic measurements. The microscopic specimen is illuminated by a monochromatic X-ray beam with a high degree of coherence, typically twice as large as the specimen’s size.

The resulting far-field diffraction pattern *I*(**q**) (**q** is the scattering vector) from an isolated sample contains high- and low-intensity interference modulations called speckles. The speckle pattern encodes information on the exact spatial distribution of the electron density *f* in the sample. Within the Born approximation, the Fourier transform 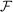 of the projected two-dimensional electron distribution is proportional to the scattered electromagnetic wavefield *F* at the detector plane in the far-field regime. Unfortunately, only the square amplitude of the wavefield is measured and the phase ϕ information, essential for the inversion, is lost. It was proposed (Sayre, 1952 ▸; Miao *et al.*, 1998 ▸) and demonstrated (Miao *et al.*, 1999 ▸) that the missing phase can be retrieved by sampling the diffraction pattern at a resolution twice higher than the Nyquist frequency.

Numerical iterative phase retrieval (IPR) algorithms have been designed (Gerchberg & Saxton, 1972 ▸; Fienup, 1987 ▸; Luke, 2004 ▸) to retrieve the missing phase and hence obtain an image of the sample via the inverse Fourier transform 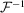 [Fig. 1 ▸(*b*)]. In this respect, the iterative algorithm can be regarded as a numerical lens. The algorithm uses the measured amplitudes 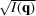 to replace the Fourier amplitudes in reciprocal space and support constraints (Fienup, 1987 ▸) in real space. Generally, the iterative reconstruction starts from a random initial guess of an object and its support. The support is the finite and compact region in real space with non-zero electron density. During the iterations, the initial support can be gradually refined; for example, by using the shrink-wrap algorithm (Marchesini *et al.*, 2003 ▸). Usually, a few thousand iterations are sufficient to achieve convergence. Typically, tens of reconstruction attempts are performed each time, starting from a random guess of the object. Only the results with the lowest error metric are retained for averaging to obtain the final reconstruction (Favre-Nicolin *et al.*, 2020*b* ▸).

There are alternative approaches for final image formation; for example, one approach uses the standard averaging method combined with an optimization procedure (Chen *et al.*, 2007 ▸). Algorithms first developed for 2D imaging can be applied to 3D data sets using a 3D fast Fourier transform (FFT). High-performance software is available for this purpose (Chapman *et al.*, 2006 ▸; Favre-Nicolin *et al.*, 2020*a* ▸). In 3D CXDI, the 3D diffraction volume is assembled from 2D diffraction patterns recorded at different sample tilts ω, taking into account the Ewald sphere curvature, thus overcoming the depth-of-focus limitations encountered in thick objects (Chapman *et al.*, 2006 ▸).

Although the IPR is a key element in CXDI, its ability to converge to an optimal solution depends on the quality of the measured diffraction patterns. Two major criteria determine the quality of the data. Firstly, the linear sampling ratio of the data σ must be σ > 2^1/*N*^, where *N* is the space dimension (Miao *et al.*, 1998 ▸); that is, the number of correlated intensities must be larger than the number of unknown variables. The experimental reality dictates that σ must be 3 or higher to ensure convergence of the IPR and reduce image artifacts (Song *et al.*, 2007 ▸). Secondly, the quantity of missing data [white areas on the detector, Fig. 1 ▸(*a*)], in particular the number of speckles in proximity to the direct beam, must be reduced to a fraction of the central speckle (Miao *et al.*, 2005 ▸). The problem in CXDI is that the direct beam must be blocked, as current detectors do not have enough dynamic range to cope with the intensity of the direct beam. Therefore, a beamstop is always used to block the direct beam, which necessarily masks the central speckles to a certain extent. Strategies to address this issue include the use of a semi-transparent beamstop (Wilke *et al.*, 2013 ▸) or the numerical mitigation of low-frequency information loss through a constrained power operator (Thibault *et al.*, 2006 ▸).

The challenge of missing data becomes more relevant for 3D CXDI. In this case, the object is rotated about one axis to collect a series of diffraction data at different object tilts ω [Fig. 1 ▸(*a*)]. The available angles for the tomographic scan are usually limited to ±80° at best, since the object is sitting on a support (*e.g.* an Si_3_N_4_ membrane) that can cast a shadow on the beam and leave an unmeasured angular interval called the missing wedge. The issue of the missing wedge becomes more pronounced when large detectors are used, as the number of pixels with unknown intensity increases cubically with the detector size. In addition, large pixel array detectors have gaps between sensor modules, which cause additional artifacts in the reconstructed images (Carnis *et al.*, 2019 ▸; Masto *et al.*, 2024 ▸). This issue can be overcome to some degree by post-processing treatments (Thibault *et al.*, 2006 ▸; Malm, 2021 ▸; Masto *et al.*, 2024 ▸; Malm & Chushkin, 2025 ▸).

The IPR works as a numerical lens and, with a sufficiently large collection angle and signal-to-noise ratio, it can out­perform the best X-ray lenses (De Andrade *et al.*, 2021 ▸) in terms of dose efficiency and resolution. In CXDI, the Abbe criterion translates into a resolution limit given by the largest scattering angles (highest spatial frequencies) where the speckles are recorded with a sufficiently good signal-to-noise ratio, and it is ultimately limited by the detector size (Skjønsfjell *et al.*, 2018*b* ▸). Since CXDI uses plane-wave illumination, small sample translations do not impact the resolution, making reconstructions resilient to mechanical instabilities. Resolution of the order of tens of nanometres has been demonstrated in several studies (Miao *et al.*, 2003 ▸; Takahashi *et al.*, 2010 ▸; Vila-Comamala *et al.*, 2011 ▸; Schropp *et al.*, 2012 ▸).

## 3. Applications

**Figure 2:**
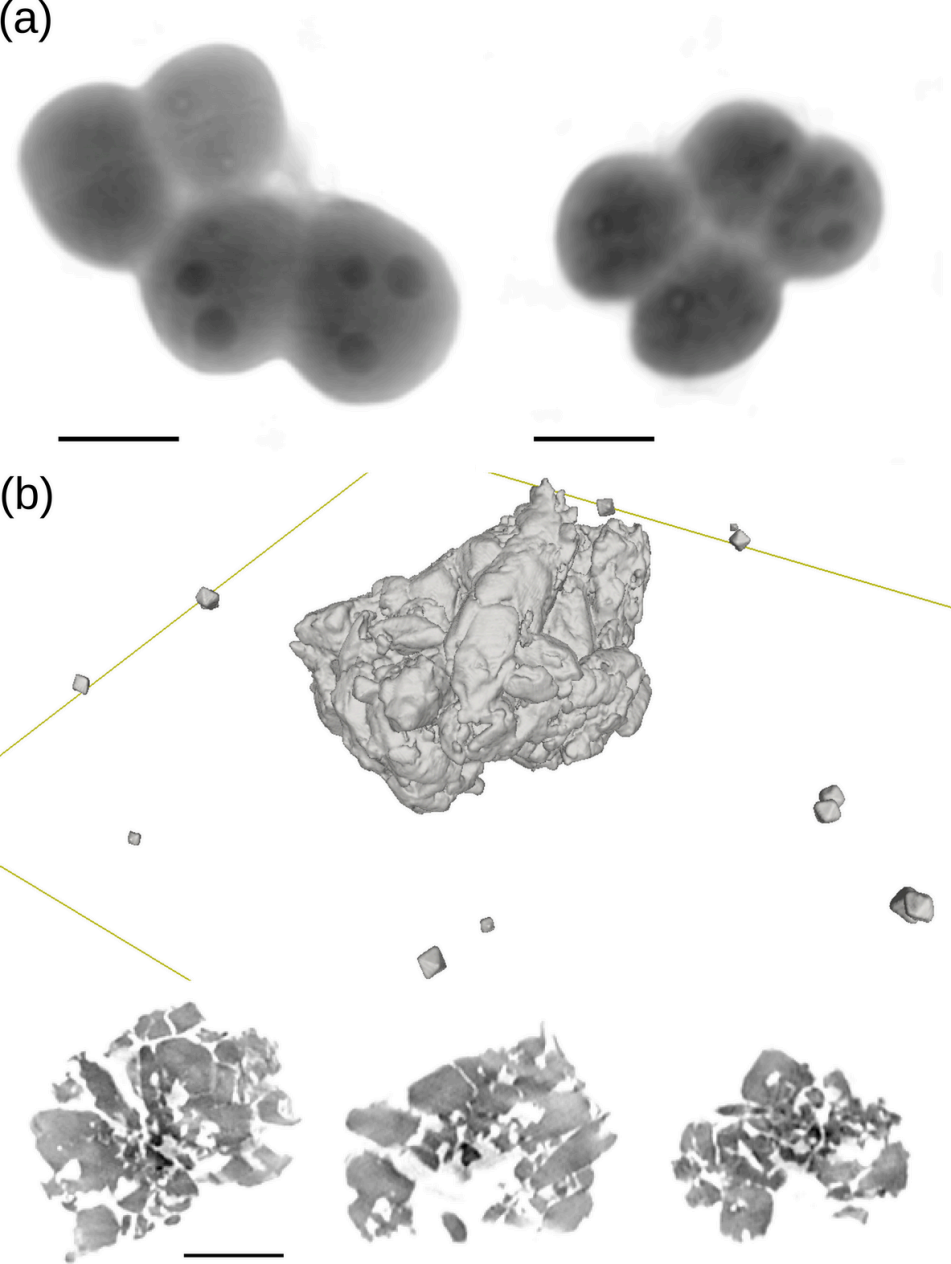
CXDI images. (*a*) A 2D projected image of *Deinococcus radiodurans* bactaria. (*b*) Three-dimensional renderings of (top) fused and individual Si nanocrystals and (bottom) central slices of a large cluster. Scale bars are 1 µm.

Various X-ray methods have been used for imaging biological objects (Dierolf *et al.*, 2010 ▸; Bartels *et al.*, 2012 ▸; Nishino *et al.*, 2012 ▸; Jacobsen, 2019 ▸). Growing interest in developing CXDI was triggered by its potential for high-resolution microscopy of biological specimens. In comparison, the application of electron microscopy commonly requires laborious sample preparation, often involving chemical staining to enhance contrast in biological specimens, in order to obtain images of thin sections with a resolution of a few ångströms (McIntosh, 2007 ▸). Here, CXDI promises to outperform previous techniques, thanks to extremely high phase sensitivity, even for specimens in a natural state. An example of *Deinococcus radiodurans* bacteria, imaged with CXDI using 8 keV radiation, is shown in Fig. 2 ▸(*a*). Unstained cells were deposited on an Si_3_N_4_ membrane, with alcohol replacing water to fix the cells. The resolution element (pixel) in these pictures is ∼32 nm and, thanks to the high sensitivity, it is possible to distinguish the cell envelope, DNA toroids, septa and dense grains without chemical staining.

There are numerous examples of CXDI being applied to biological imaging. Early work by Jiang *et al.* (2008 ▸) demonstrated the application of CXDI for imaging biominerals in fish bone and in bovine bones (Verezhak, 2016 ▸) at nanometre resolution. Subsequent imaging of a whole unstained *Schizosaccharomyces pombe* yeast spore cell using chemical fixation (Jiang *et al.*, 2010 ▸) revealed the 3D morphology and structure of cell organelles at 50–60 nm resolution. The cell structure can be preserved in a close-to-natural state using cryogenic freezing. Freeze-plunging is widely used in X-ray crystallography, and in electron and X-ray microscopy. For example, it was applied for imaging frozen-hydrated *D. radiodurans* bacteria (Lima *et al.*, 2009 ▸), a *Neospora caninum* cell (Rodriguez *et al.*, 2015 ▸) and near-surface structures of frozen-hydrated malaria-infected human erythrocytes (Frank *et al.*, 2017 ▸). Cryogenic cooling is also a standard approach for mitigating radiation damage of biological specimens, which is a major limitation for achieving high resolution. This damage can result in mass loss, as demonstrated in metal-coated polymeric spheres (Skjønsfjell *et al.*, 2018*a* ▸). Extrapolating from experimental data and theoretical calculations, Howells and co-workers estimated that biological imaging at 10 nm resolution should be achievable using cryogenic cooling (Howells *et al.*, 2009 ▸), yet it still remains to be demonstrated.

The unique capabilities of CXDI are particularly well suited for imaging materials science specimens, which are generally more resistant to radiation damage. Fig. 2 ▸(*b*) shows CXDI images of a cluster of Si nanoparticles deposited on an Si_3_N_4_ membrane. The cluster contains individual octahedral nanoparticles, each ∼200 nm in size, present on both the surface of the membrane and within the cluster. The central large cluster has a porous internal morphology, which makes such materials interesting for optoelectronics applications (de Jong *et al.*, 2016 ▸). CXDI has also been used to investigate TiO_2_ sponges infiltrated with a perovskite for photovoltaic applications (Sanzaro *et al.*, 2019 ▸).

CXDI has also been used to study complex biomineral architectures, such as the exoskeleton structure of coccolithophores, known as coccospheres (Beuvier *et al.*, 2019 ▸). A coccosphere consists of an assembly of imbricated coccoliths, composed of calcite crystals produced by the cells. The strong scattering power of the CaCO_3_ specimens, along with their robustness to X-rays, enabled high-quality 3D imaging. This revealed correlations between coccolith mass and grid size, as well as size variability within a coccosphere (Beuvier *et al.*, 2019 ▸).

CaCO_3_, a widespread product of biomineralization by living organisms, is ubiquitous in nature. As a light and biocompatible substance, it has found numerous industrial applications and can be used as a model system to study biomineralization and non-classical nucleation and growth mechanisms (De Yoreo *et al.*, 2015 ▸). CaCO_3_ appears in three polymorph crystalline phases: calcite, aragonite and vaterite, all of which can be synthesized in a laboratory under specific conditions of temperature, concentration, additive presence and time. CXDI is an indispensable tool to visualize the 3D internal porosity of CaCO_3_ microparticles. The method was used to follow the modification of pore geometry during the solid-state transformation of vaterite spherical particles to calcite (Cherkas *et al.*, 2017 ▸) during high-temperature annealing. Another study investigated the phase-transformation kinetics of dried vaterite particles immersed in deionized and tap water (Cherkas *et al.*, 2018 ▸). Recently, a comprehensive study uncovered the mechanism that governs the formation of hollow vaterite microparticles (Beuvier *et al.*, 2022 ▸). The particles were synthesized by varying the ion concentration and the amount of an additive (polystyrene sulfonate, PSS) that slows down the kinetics of the process. The specimens were collected as a function of reaction time to follow the time evolution of the particle morphology. The work showed that Ostwald ripening cannot explain the observed diversity of crystal sizes, shapes and spatial organization. For this, other factors, such as PSS concentration and dipole–dipole inter­actions, must be accounted for in order to explain the transformation from a whole to a hollow particle (Beuvier *et al.*, 2022 ▸). In order to resolve the crystalline phases of CaCO_3_ or other single-particle minerals (Chattopadhyay *et al.*, 2020 ▸), combined CXDI and Bragg diffraction measurements were developed (Chushkin *et al.*, 2019 ▸).

These selected examples demonstrate the power of CXDI at third-generation synchrotrons. Nevertheless, new-generation synchrotron sources are expected to boost the capabilities of the method significantly.

## 4. Fourth-generation synchrotron sources

Fourth-generation synchrotron sources are based on the multi-bend achromat (MBA) lattice design (Einfeld *et al.*, 1995 ▸), which reduces the horizontal emittance by more than an order of magnitude, producing X-ray beams with a high degree of coherence (Raimondi *et al.*, 2023 ▸). The MBA lattice has been exploited to construct new synchrotron storage rings, such as Max IV and Sirius, but it can also be efficiently implemented at third-generation synchrotrons through the hybrid MBA design (Biasci *et al.*, 2014 ▸; ESRF, 2014 ▸), as demonstrated by the ESRF’s Extremely Brilliant Source (ESRF-EBS) (Raimondi *et al.*, 2023 ▸). This approach paves the way for upgrades of existing third-generation synchrotrons worldwide.

**Figure 3:**
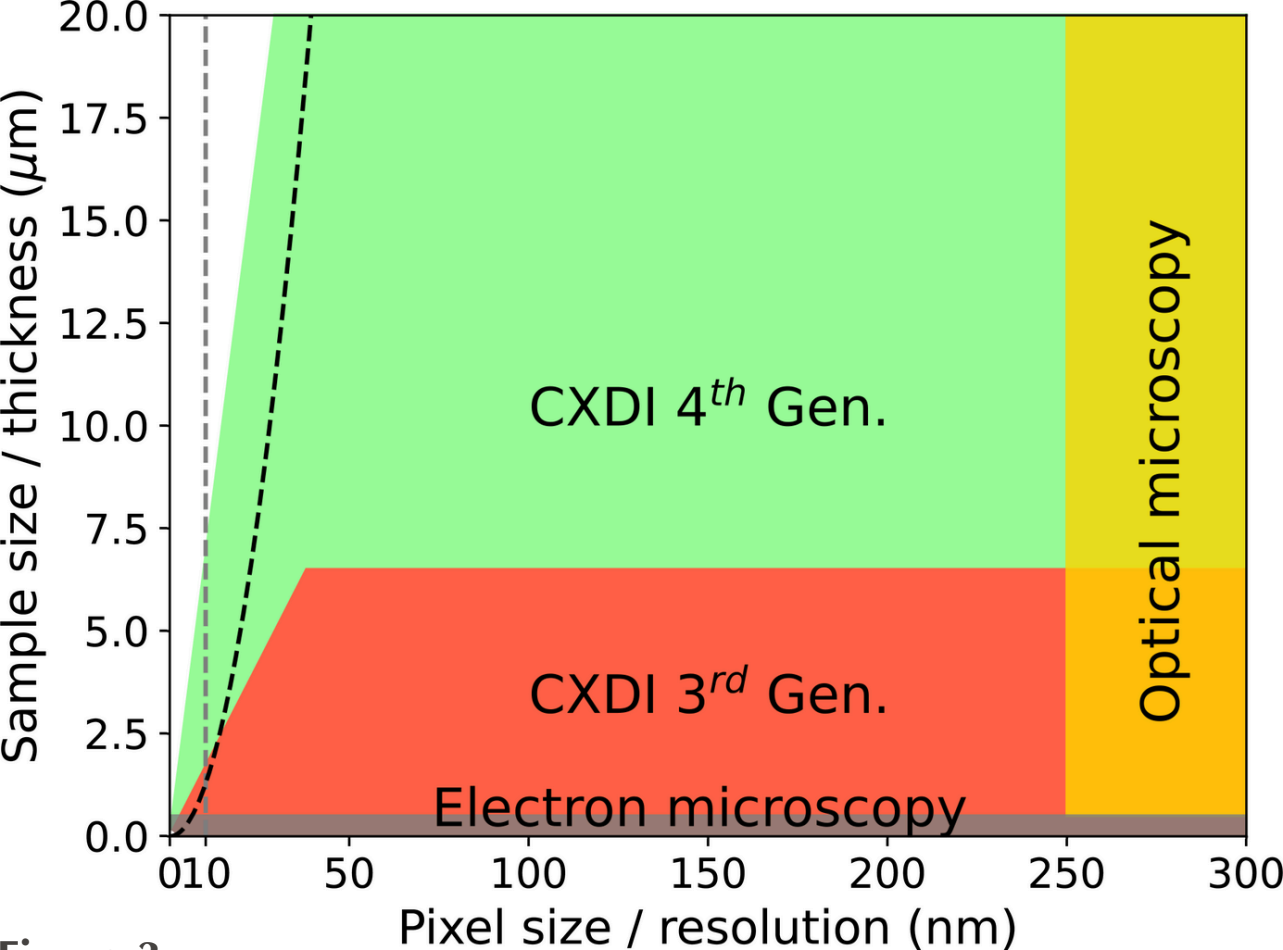
Sample size and resolution coverage of CXDI, electron and optical microscopy techniques. The gray vertical line is the 10 nm resolution limit and the black dashed line defines the DOF boundary at λ = 1.55 Å.

High brilliance and low emittance are essential for coherent scattering techniques, as transverse coherence length scales inversely with emittance, while coherent flux is proportional to brilliance. For CXDI, such properties result in a larger field of view (object size), faster data collection and higher image resolution. The large spatial coherence enables the imaging of larger objects, in particular biological cells and materials of approximately 10–20 µm in size. Generally, biological cells are studied either with conventional optical microscopy, which can provide images of transparent specimens at a resolution of hundreds of nanometres (250 nm in Fig. 3 ▸), or with electron microscopy, which provides atomic scale resolution but only of thin sections a few hundred nanometres (250 nm) thick (Fig. 3 ▸). X-ray microscopy, in particular CXDI, at highly brilliant sources bridges the gap between these two techniques by imaging specimens a few micrometres thick at nanometre resolution.

Fig. 3 ▸ summarizes the expanded capabilities of CXDI with a fourth-generation synchrotron source. The red area delineates the accessible region at third-generation sources. The maximum sample size *S* is limited by the transverse coherence length and available sample-to-detector distance *D*, which follows the linear sampling ratio σ = *D*λ/*pS* = 3, where λ is the wavelength and *p* is the detector pixel size. The slope of the region defining the resolution element Δ*r* is given by the sample size *S* and the detector size in pixels *N*_pix_, assuming sufficient signal is recorded at the highest frequencies and maintaining a fixed sampling ratio of 3, giving Δ*r* = 3 × *S*/*N*_pix_. Following the assumption of Porod’s law, that the average speckle intensity *I*(**q**) falls as *I*(**q**) ≃ **q**^−4^, a hundredfold increase in brilliance results in a resolution gain of about 100^1/4^ ≃ 3 for the same signal-to-noise ratio. The improvement expected with fourth-generation sources and a detector four times larger is represented by the green region in Fig. 3 ▸. For our calculations, we assume a detector with 2048 × 2048 pixels and a pixel size *p* = 75 µm positioned 30 m from a sample illuminated by a coherent 8 keV plane wave. Under such conditions, imaging samples up to 20 µm in size at (sub-) 30 nm resolution should make CXDI even more attractive for biologists, as many interesting biological specimens fall within this size range.

**Figure 4:**
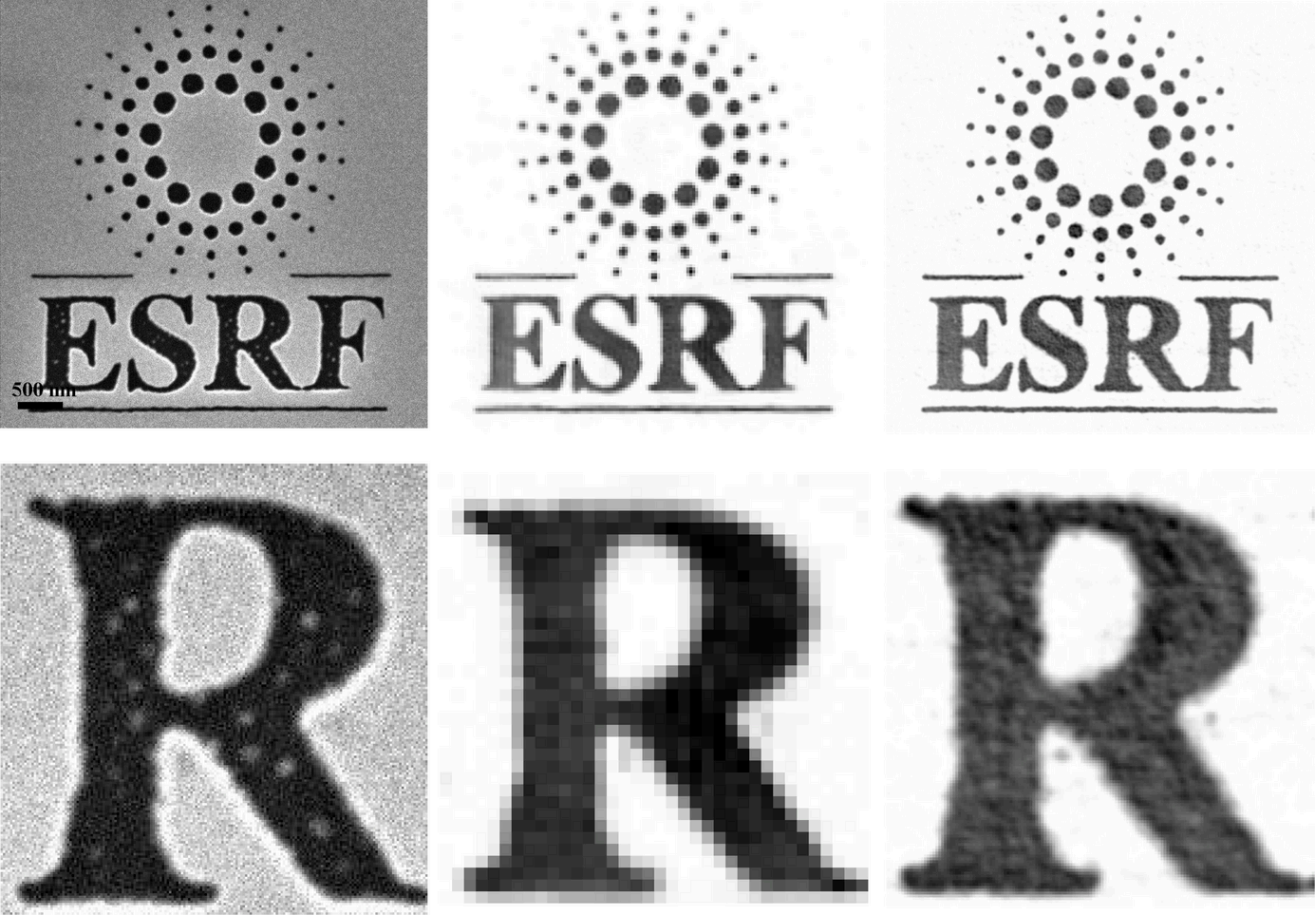
(Top row) A 5 µm ESRF logo test sample imaged by (left) SEM, (middle) CXDI before the EBS at 32.5 nm pixel size and (right) with the EBS at 7.6 nm pixel size. (Bottom row) Enlargements of the letter ‘R’ demonstrate the increase in resolution.

We note that samples made of radiation-hard materials that are smaller than 7 µm can be routinely imaged at sub-10 nm resolution. As an example, Fig. 4 ▸ shows the capability of the new ESRF-EBS coupled with a large detector to provide a resolution of below 10 nm of a test logo sample patterned in a 216 nm thin tungsten layer. It is important to note that the sample thickness is a parameter that can constrain the resolution of an image. When the sample thickness exceeds the depth of field (DOF), given by DOF = 2Δ*r*^2^/λ, the resulting 2D image exhibits defocus artifacts (Chapman *et al.*, 2006 ▸). The DOF boundary, defined for a wavelength of λ = 1.55 Å, is shown in Fig. 3 ▸ and is more restrictive than the detector size constraints (green area). Using shorter wavelengths shifts the boundary towards higher resolution, but it also reduces the sample size/thickness for which oversampling can be achieved within the available sample-to-detector distance. Nevertheless, alternative approaches can be applied. In ptychography, the defocus artifacts in 2D imaging can potentially be solved numerically using the multi-slice method (Maiden *et al.*, 2012 ▸; Tsai *et al.*, 2016 ▸). In CXDI, the issue is resolved by taking the Ewald sphere curvature into account when building the 3D diffraction volume (Chapman *et al.*, 2006 ▸) or a 2D slice (Takahashi *et al.*, 2010 ▸) from the 2D diffraction patterns measured at different sample tilts. It is important to note that other X-ray imaging techniques can cover certain portions of the sample size domain shown in Fig. 3 ▸, but CXDI holds the potential to achieve the finest resolution.

Coherent X-rays can also be used for imaging magnetic nanostructures and their dynamics with soft X-rays (Eisebitt *et al.*, 2004 ▸; Flewett *et al.*, 2012 ▸; Ukleev *et al.*, 2018 ▸) and, recently, with hard X-rays (Donnelly *et al.*, 2017 ▸) to probe magnetic domains, domain walls, vortexes and skyrmions. Hard X-rays, in the range of 6–12 keV, are necessary for imaging microscopic specimens that are too thick (>400 nm) for soft X-rays, but magnetic contrast in this energy range is rather low (Donnelly *et al.*, 2016 ▸). Thus, the high coherent flux provided by fourth-generation sources is mandatory for exploiting hard X-rays to characterize magnetic textures in materials (Donnelly & Scagnoli, 2020 ▸).

One of the little-exploited but high-potential applications of CXDI is time-resolved imaging to study dynamic phenomena (Grote *et al.*, 2022 ▸). Due to its non-scanning nature, fast recording (kilohertz frame rate) of diffraction data is possible with current state-of-the-art pixel array detectors. Plane-wave illumination, used in CXDI, provides robustness to any unwanted sample nano-translations and ensures high resolution. Real-time imaging of dynamic phenomena was demonstrated in a proof-of-principle experiment using a coherent optical light source (Lo *et al.*, 2018 ▸). In the hard X-ray regime, the combination of X-ray photon correlation spectroscopy with CXDI for time-resolved imaging was recently proposed (Takazawa *et al.*, 2023 ▸; Hinsley *et al.*, 2024 ▸). Rapid data collection, combined with analysis of photon-sparse random projections (Ayyer *et al.*, 2016 ▸; Giewekemeyer *et al.*, 2019 ▸), can be useful in applications with optical trapping and manipulation (Gao *et al.*, 2019 ▸) or levitating specimens. Sample levitation/manipulation in air or liquid (Kolb *et al.*, 2015 ▸; Müller *et al.*, 2015 ▸) could be exploited for random sample rotations in order to generate sufficient data for a complete 3D diffraction volume without the missing wedge. The challenge of constructing a 3D diffraction volume with sufficient statistics from random sample orientations is central in single-particle imaging at XFELs, and a solution has been developed by Loh & Elser (2009 ▸). Recent studies have demonstrated significant progress by reconstructing 3D structures of viruses (Rose *et al.*, 2018 ▸) and nanoparticles (Ayyer *et al.*, 2021 ▸; Nakano *et al.*, 2022 ▸) from a series of single-shot diffraction patterns at an XFEL. The proposed solutions for data sorting, background subtraction, sample handling and image reconstruction can be applied directly to synchrotron CXDI imaging. For example, the expand–maximize–compress algorithm (Loh & Elser, 2009 ▸) for assembling 3D diffraction volumes from random sample orientations was adopted for Bragg CXDI (Björling *et al.*, 2020 ▸) to manage uncontrolled particle rotations during measurements.

To capitalize fully on the new possibilities offered by fourth-generation synchrotron sources, specialized instruments (Chushkin *et al.*, 2014 ▸; Johansson *et al.*, 2021 ▸) must be developed, alongside advancements in detectors (Grimes *et al.*, 2023 ▸; Takahashi *et al.*, 2023 ▸), to enhance detection efficiency for both high- and low-intensity signals.

Further development of CXDI should focus on improving the performance of the numerical lens. Efforts should be directed towards improving algorithm robustness to noise, handling sparse data sets, reducing artifacts caused by missing information and overcoming depth-of-focus limitation in thick samples to achieve the ultimate resolution. For example, the new general proximal smoothing algorithm (Pham *et al.*, 2019 ▸) outperforms classical algorithms when phasing noisy data. The latest phase retrieval developments incorporate the total variation regularization to compensate for missing data (Chang *et al.*, 2016 ▸) and demonstrate efficiency in imaging magnetic domains (Yokoyama *et al.*, 2022 ▸).

A promising approach is to employ machine learning methods (Harder, 2021 ▸). There are numerous examples of the effectiveness of machine learning for IPR. The introduction of data treatments based on neural networks is useful for recovering missing data and reducing noise (Bellisario *et al.*, 2022 ▸), as well as for removing artifacts caused by the detector gaps (Masto *et al.*, 2024 ▸). Deep neural network models are also effective for complex single-particle imaging (Wu *et al.*, 2021 ▸). Additionally, machine learning can be used to find reliable object support (Shang *et al.*, 2025 ▸). New approaches for resolution enhancement via the extrapolation of experimental data could also be effective in some cases (Latychevskaia *et al.*, 2015 ▸).

## 5. Conclusions

After 25 years of existence, coherent X-ray imaging (CXDI) has proved to be a very powerful method for imaging bio­logical and materials science specimens on the nanoscale. A wide range of objects, from biological cells, bacteria and bones to the exoskeletons of algae, minerals, mesostructures and porous particles for electronics have been scrutinized using CXDI.

At third-generation synchrotrons, CXDI has occupied a niche for visualizing the 3D structure of naturally microscopic and isolated specimens. Typically, the largest size of the investigated objects was limited to ∼6–7 µm, due to the small transverse coherence length of the beam, and with a resolution of around 15–30 nm, due to the size of the detector and the coherent flux. The new generation of synchrotron sources with highly coherent X-ray beams will enhance the capability of CXDI to image 10–20 µm specimens with even greater detail and in a shorter time. Such improvements result in a better tool for visualizing cells, bone fragments, exoskeletons of marine algae and micrometre-sized nanostructures, as well as enhanced capabilities for time-resolved studies of the formation of crystalline microparticles, biominerals *etc.*

To benefit fully from these new opportunities, further development of instrumentation and detectors is necessary, along with numerical algorithms for phase retrieval, noise reduction and ultimate resolution.

## References

[bb1] Aidukas, T., Phillips, N. W., Diaz, A., Poghosyan, E., Müller, E., Levi, A. F. J., Aeppli, G., Guizar-Sicairos, M. & Holler, M. (2024). *Nature*, **632**, 81–88. 10.1038/s41586-024-07615-6 39085541

[bb2] Ayyer, K., Lan, T.-Y., Elser, V. & Loh, N. D. (2016). *J. Appl. Cryst.* **49**, 1320–1335. 10.1107/S1600576716008165 PMC4970497 27504078

[bb3] Ayyer, K., Xavier, P. L., Bielecki, J., Shen, Z., Daurer, B. J., Samanta, A. K., Awel, S., Bean, R., Barty, A., Bergemann, M., Ekeberg, T., Estillore, A. D., Fangohr, H., Giewekemeyer, K., Hunter, M. S., Karnevskiy, M., Kirian, R. A., Kirkwood, H., Kim, Y., Koliyadu, J., Lange, H., Letrun, R., Lübke, J., Michelat, T., Morgan, A. J., Roth, N., Sato, T., Sikorski, M., Schulz, F., Spence, J. C. H., Vagovic, P., Wollweber, T., Worbs, L., Yefanov, O., Zhuang, Y., Maia, F. R. N. C., Horke, D. A., Küpper, J., Loh, N. D., Mancuso, A. P. & Chapman, H. N. (2021). *Optica*, **8**, 15–23.

[bb4] Bajt, S., Prasciolu, M., Fleckenstein, H., Domaracký, M., Chapman, H. N., Morgan, A. J., Yefanov, O., Messerschmidt, M., Du, Y., Murray, K. T., Mariani, V., Kuhn, M., Aplin, S., Pande, K., Villanueva-Perez, P., Stachnik, K., Chen, J. P., Andrejczuk, A., Meents, A., Burkhardt, A., Pennicard, D., Huang, X., Yan, H., Nazaretski, E., Chu, Y. S. & Hamm, C. E. (2018). *Light Sci. Appl.* **7**, 17162–17162. 10.1038/lsa.2017.162 PMC6060042 30839543

[bb5] Bartels, M., Priebe, M., Wilke, R. N., Krüger, S. P., Giewekemeyer, K., Kalbfleisch, S., Olendrowitz, C., Sprung, M. & Salditt, T. (2012). *Opt. Nanoscopy*, **1**, 10.

[bb6] Baruchel, J., Bleuet, P., Bravin, A., Coan, P., Lima, E., Madsen, A., Ludwig, W., Pernot, P. & Susini, J. (2008). *C. R. Phys.* **9**, 624–641.

[bb7] Bellisario, A., Maia, F. R. N. C. & Ekeberg, T. (2022). *J. Appl. Cryst.* **55**, 122–132. 10.1107/S1600576721012371 PMC8805166 35145358

[bb8] Bérujon, S., Ziegler, E., Cerbino, R. & Peverini, L. (2012). *Phys. Rev. Lett.* **108**, 158102. 10.1103/PhysRevLett.108.158102 22587288

[bb9] Beuvier, T., Chushkin, Y., Zontone, F., Gibaud, A., Cherkas, O., Da Silva, J. & Snigireva, I. (2022). *IUCrJ*, **9**, 580–593. 10.1107/S2052252522006108 PMC9438498 36071800

[bb10] Beuvier, T., Probert, I., Beaufort, L., Suchéras-Marx, B., Chushkin, Y., Zontone, F. & Gibaud, A. (2019). *Nat. Commun.* **10**, 751. 10.1038/s41467-019-08635-x PMC6375944 30765698

[bb11] Biasci, J. C., Bouteille, J. F., Carmignani, N., Chavanne, J., Coulon, D., Dabin, Y., Ewald, F., Farvacque, L., Goirand, L., Hahn, M., Jacob, J., LeBec, G., Liuzzo, S., Nash, B., Pedroso-Marques, H., Perron, T., Plouviez, E., Raimondi, P., Revol, J. L., Scheidt, K. & Serrière, V. (2014). *Synchrotron Radiation News*, **27**(6), 8–12.

[bb12] Björling, A., Marçal, L. A. B., Solla-Gullón, J., Wallentin, J., Carbone, D. & Maia, F. R. N. C. (2020). *Phys. Rev. Lett.* **125**, 246101. 10.1103/PhysRevLett.125.246101 33412038

[bb13] Carnis, J., Gao, L., Labat, S., Kim, Y. Y., Hofmann, J. P., Leake, S. J., Schülli, T. U., Hensen, E. J. M., Thomas, O. & Richard, M.-I. (2019). *Sci. Rep.* **9**, 17357. 10.1038/s41598-019-53774-2 PMC6874548 31758040

[bb14] Chang, H., Lou, Y., Ng, M. K. & Zeng, T. (2016). *SIAM J. Sci. Comput.* **38**, A3672–A3695.

[bb15] Chao, W., Harteneck, B. D., Liddle, J. A., Anderson, E. H. & Attwood, D. T. (2005). *Nature*, **435**, 1210–1213. 10.1038/nature03719 15988520

[bb16] Chapman, H. N., Barty, A., Marchesini, S., Noy, A., Hau-Riege, S. P., Cui, C., Howells, M. R., Rosen, R., He, H., Spence, J. C. H., Weierstall, U., Beetz, T., Jacobsen, C. & Shapiro, D. (2006). *J. Opt. Soc. Am. A*, **23**, 1179–1200. 10.1364/josaa.23.001179 16642197

[bb17] Chapman, H. N. & Nugent, K. A. (2010). *Nat. Photon.* **4**, 833–839.

[bb18] Chattopadhyay, B., Madathiparambil, A. S., Mürer, F. K., Cerasi, P., Chushkin, Y., Zontone, F., Gibaud, A. & Breiby, D. W. (2020). *J. Appl. Cryst.* **53**, 1562–1569. 10.1107/S1600576720013850 PMC7710485 33304225

[bb19] Chen, C.-C., Miao, J., Wang, C. W. & Lee, T. K. (2007). *Phys. Rev. B*, **76**, 064113.

[bb20] Cherkas, O., Beuvier, T., Breiby, D. W., Chushkin, Y., Zontone, F. & Gibaud, A. (2017). *Cryst. Growth Des.* **17**, 4183–4188.

[bb21] Cherkas, O., Beuvier, T., Zontone, F., Chushkin, Y., Demoulin, L., Rousseau, A. & Gibaud, A. (2018). *Adv. Powder Technol.* **29**, 2872–2880.

[bb22] Chushkin, Y., Zontone, F., Cherkas, O. & Gibaud, A. (2019). *J. Appl. Cryst.* **52**, 571–578.

[bb23] Chushkin, Y., Zontone, F., Lima, E., De Caro, L., Guardia, P., Manna, L. & Giannini, C. (2014). *J. Synchrotron Rad.* **21**, 594–599. 10.1107/S1600577514003440 24763650

[bb24] Cloetens, P., Ludwig, W., Baruchel, J., Van Dyck, D., Van Landuyt, J., Guigay, J. P. & Schlenker, M. (1999). *Appl. Phys. Lett.* **75**, 2912–2914.

[bb25] De Andrade, V., Nikitin, V., Wojcik, M., Deriy, A., Bean, S., Shu, D., Mooney, T., Peterson, K., Kc, P., Li, K., Ali, S., Fezzaa, K., Gürsoy, D., Arico, C., Ouendi, S., Troadec, D., Simon, P., De Carlo, F. & Lethien, C. (2021). *Adv. Mater.* **33**, 2008653. 10.1002/adma.202008653 33871108

[bb26] Dierolf, M., Menzel, A., Thibault, P., Schneider, P., Kewish, C. M., Wepf, R., Bunk, O. & Pfeiffer, F. (2010). *Nature*, **467**, 436–439. 10.1038/nature09419 20864997

[bb27] Donnelly, C., Guizar-Sicairos, M., Scagnoli, V., Gliga, S., Holler, M., Raabe, J. & Heyderman, L. J. (2017). *Nature*, **547**, 328–331. 10.1038/nature23006 28726832

[bb28] Donnelly, C. & Scagnoli, V. (2020). *J. Phys. Condens. Matter*, **32**, 213001. 10.1088/1361-648X/ab5e3c 31796657

[bb29] Donnelly, C., Scagnoli, V., Guizar-Sicairos, M., Holler, M., Wilhelm, F., Guillou, F., Rogalev, A., Detlefs, C., Menzel, A., Raabe, J. & Heyderman, L. J. (2016). *Phys. Rev. B*, **94**, 064421.

[bb30] Einfeld, D., Schaper, J. & Plesko, M. (1995). *Proceedings Particle Accelerator Conference*, Dallas, TX, USA, Vol. 1, pp. 177–179.

[bb31] Eisebitt, S., Lüning, J., Schlotter, W. F., Lörgen, M., Hellwig, O., Eberhardt, W. & Stöhr, J. (2004). *Nature*, **432**, 885–888. 10.1038/nature03139 15602557

[bb74] ESRF (2014). *ESRF Upgrade Programme Phase II (2015–2022), Technical Design Study*. http:/www.esrf.fr/Apache_files/Upgrade/ESRF-orange-book.pdf.

[bb32] Favre-Nicolin, V., Girard, G., Leake, S., Carnis, J., Chushkin, Y., Kieffer, J., Paleo, P. & Richard, M.-I. (2020*a*). *J. Appl. Cryst.* **53**, 1404–1413.

[bb33] Favre-Nicolin, V., Leake, S. & Chushkin, Y. (2020*b*). *Sci. Rep.* **10**, 2664. 10.1038/s41598-020-57561-2 PMC7021796 32060293

[bb34] Fienup, J. R. (1987). *J. Opt. Soc. Am. A*, **4**, 118–123.

[bb35] Flewett, S., Schaffert, S., Mohanty, J., Guehrs, E., Geilhufe, J., Günther, C. M., Pfau, B. & Eisebitt, S. (2012). *Phys. Rev. Lett.* **108**, 223902. 10.1103/PhysRevLett.108.223902 23003595

[bb36] Frank, V., Chushkin, Y., Fröhlich, B., Abuillan, W., Rieger, H., Becker, A. S., Yamamoto, A., Rossetti, F. F., Kaufmann, S., Lanzer, M., Zontone, F. & Tanaka, M. (2017). *Sci. Rep.* **7**, 14081. 10.1038/s41598-017-14586-4 PMC5658481 29074975

[bb37] Gao, Y., Harder, R., Southworth, S. H., Guest, J. R., Huang, X., Yan, Z., Ocola, L. E., Yifat, Y., Sule, N., Ho, P. J., Pelton, M., Scherer, N. F. & Young, L. (2019). *Proc. Natl Acad. Sci. USA*, **116**, 4018–4024. 10.1073/pnas.1720785116 PMC6410780 30765527

[bb38] Gerchberg, R. W. & Saxton, W. O. (1972). *Optik (Stuttgart)*, **35**, 237–246.

[bb39] Giewekemeyer, K., Aquila, A., Loh, N.-T. D., Chushkin, Y., Shanks, K. S., Weiss, J. T., Tate, M. W., Philipp, H. T., Stern, S., Vagovic, P., Mehrjoo, M., Teo, C., Barthelmess, M., Zontone, F., Chang, C., Tiberio, R. C., Sakdinawat, A., Williams, G. J., Gruner, S. M. & Mancuso, A. P. (2019). *IUCrJ*, **6**, 357–365. 10.1107/S2052252519002781 PMC6503918 31098017

[bb40] Grimes, M., Pauwels, K., Schülli, T. U., Martin, T., Fajardo, P., Douissard, P.-A., Kocsis, M., Nishino, H., Ozaki, K., Honjo, Y., Nishiyama Hiraki, T., Joti, Y., Hatsui, T., Levi, M., Rabkin, E., Leake, S. J. & Richard, M.-I. (2023). *J. Appl. Cryst.* **56**, 1032–1037. 10.1107/S1600576723004314 PMC10405578 37555222

[bb41] Grote, L., Seyrich, M., Döhrmann, R., Harouna-Mayer, S. Y., Mancini, F., Kaziukenas, E., Fernandez-Cuesta, I. A., Zito, C., Vasylieva, O., Wittwer, F., Odstrčzil, M., Mogos, N., Landmann, M., Schroer, C. G. & Koziej, D. (2022). *Nat. Commun.* **13**, 4971. 10.1038/s41467-022-32373-2 PMC9424245 36038564

[bb42] Harder, R. (2021). *IUCrJ*, **8**, 1–3. 10.1107/S2052252520016590 PMC7793000 33520237

[bb43] Hinsley, G. N., Westermeier, F., Wang, B., Ngoi, K. H., Singh, S., Rysov, R., Sprung, M., Kewish, C. M., van Riessen, G. A. & Vartanyants, I. A. (2024). *Nano Lett.* **24**, 13702–13707. 10.1021/acs.nanolett.4c03699 PMC11528431 39423316

[bb44] Holler, M., Guizar-Sicairos, M., Tsai, E. H. R., Dinapoli, R., Müller, E., Bunk, O., Raabe, J. & Aeppli, G. (2017). *Nature*, **543**, 402–406. 10.1038/nature21698 28300088

[bb45] Howells, M., Beetz, T., Chapman, H., Cui, C., Holton, J., Jacobsen, C., Kirz, J., Lima, E., Marchesini, S., Miao, H., Sayre, D., Shapiro, D., Spence, J. & Starodub, D. (2009). *J. Electron Spectrosc. Relat. Phenom.* **170**, 4–12. 10.1016/j.elspec.2008.10.008 PMC2867487 20463854

[bb46] Jacobsen, C. (2019). *X-ray Microscopy.* Cambridge University Press.

[bb47] Jiang, H., Ramunno-Johnson, D., Song, C., Amirbekian, B., Kohmura, Y., Nishino, Y., Takahashi, Y., Ishikawa, T. & Miao, J. (2008). *Phys. Rev. Lett.* **100**, 038103. 10.1103/PhysRevLett.100.038103 18233041

[bb48] Jiang, H., Song, C., Chen, C.-C., Xu, R., Raines, K. S., Fahimian, B. P., Lu, C.-H., Lee, T.-K., Nakashima, A., Urano, J., Ishikawa, T., Tamanoi, F. & Miao, J. (2010). *Proc. Natl Acad. Sci. USA*, **107**, 11234–11239. 10.1073/pnas.1000156107 PMC2895086 20534442

[bb49] Johansson, U., Carbone, D., Kalbfleisch, S., Björling, A., Kahnt, M., Sala, S., Stankevic, T., Liebi, M., Rodriguez Fernandez, A., Bring, B., Paterson, D., Thånell, K., Bell, P., Erb, D., Weninger, C., Matej, Z., Roslund, L., Åhnberg, K., Norsk Jensen, B., Tarawneh, H., Mikkelsen, A. & Vogt, U. (2021). *J. Synchrotron Rad.* **28**, 1935–1947. 10.1107/S1600577521008213 PMC8570223 34738949

[bb50] Jong, E. M. L. D. de, Mannino, G., Alberti, A., Ruggeri, R., Italia, M., Zontone, F., Chushkin, Y., Pennisi, A. R., Gregorkiewicz, T. & Faraci, G. (2016). *Sci. Rep.* **6**, 25664. 10.1038/srep25664 PMC4877587 27216452

[bb51] Kalbfleisch, S., Zhang, Y., Kahnt, M., Buakor, K., Langer, M., Dreier, T., Dierks, H., Stjärneblad, P., Larsson, E., Gordeyeva, K., Chayanun, L., Söderberg, D., Wallentin, J., Bech, M. & Villanueva-Perez, P. (2022). *J. Synchrotron Rad.* **29**, 224–229. 10.1107/S1600577521012200 PMC8733976 34985439

[bb52] Kolb, T., Albert, S., Haug, M. & Whyte, G. (2015). *J. Biophotonics*, **8**, 239–246. 10.1002/jbio.201300196 24733809

[bb53] Latychevskaia, T., Chushkin, Y., Zontone, F. & Fink, H.-W. (2015). *Appl. Phys. Lett.* **107**, 183102.

[bb54] Lima, E., Wiegart, L., Pernot, P., Howells, M., Timmins, J., Zontone, F. & Madsen, A. (2009). *Phys. Rev. Lett.* **103**, 198102. 10.1103/PhysRevLett.103.198102 20365956

[bb55] Lo, Y. H., Zhao, L., Gallagher-Jones, M., Rana, A. J., Lodico, J., Xiao, W., Regan, B. C. & Miao, J. (2018). *Nat. Commun.* **9**, 1826. 10.1038/s41467-018-04259-9 PMC5940918 29739941

[bb56] Loh, N. D. & Elser, V. (2009). *Phys. Rev. E*, **80**, 026705. 10.1103/PhysRevE.80.026705 19792279

[bb57] Luke, D. R. (2005). *Inverse Probl.* **21**, 37–50.

[bb58] Maiden, A. M., Humphry, M. J. & Rodenburg, J. M. (2012). *J. Opt. Soc. Am. A*, **29**, 1606–1614. 10.1364/JOSAA.29.001606 23201876

[bb59] Malm, E. (2021). PhD thesis, Lund University, Sweden.

[bb60] Malm, E. & Chushkin, Y. (2025). *J. Synchrotron Rad.* **32**, 210–216. 10.1107/S1600577524010956 PMC11708845 39700021

[bb61] Marchesini, S., He, H., Chapman, H. N., Hau-Riege, S. P., Noy, A., Howells, M. R., Weierstall, U. & Spence, J. C. H. (2003). *Phys. Rev. B*, **68**, 140101. 10.1364/oe.11.002344 19471343

[bb62] Masto, M., Favre-Nicolin, V., Leake, S., Schülli, T., Richard, M.-I. & Bellec, E. (2024). *J. Appl. Cryst.* **57**, 966–974. 10.1107/S1600576724004163 PMC11299604 39108812

[bb63] McIntosh, J. R. (2007). *Cellular Electron Microscopy*, edited by J. R. McIntosh, pp. xxi–xxvii. Academic Press.

[bb64] McNulty, I., Kirz, J., Jacobsen, C., Anderson, E. H., Howells, M. R. & Kern, D. P. (1992). *Science*, **256**, 1009–1012. 10.1126/science.256.5059.1009 17795006

[bb65] Miao, J., Charalambous, P., Kirz, J. & Sayre, D. (1999). *Nature*, **400**, 342–344.

[bb66] Miao, J., Ishikawa, T., Anderson, E. H. & Hodgson, K. O. (2003). *Phys. Rev. B*, **67**, 174104.

[bb67] Miao, J., Nishino, Y., Kohmura, Y., Johnson, B., Song, C., Risbud, S. H. & Ishikawa, T. (2005). *Phys. Rev. Lett.* **95**, 085503. 10.1103/PhysRevLett.95.085503 16196870

[bb68] Miao, J., Sayre, D. & Chapman, H. N. (1998). *J. Opt. Soc. Am. A*, **15**, 1662–1669.

[bb69] Mokso, R., Cloetens, P., Maire, E., Ludwig, W. & Buffière, J.-Y. (2007). *Appl. Phys. Lett.* **90**, 144104.

[bb70] Müller, P., Schürmann, M., Chan, C. J. & Guck, J. (2015). *Proc. SPIE*, **9548**, 95480U.

[bb71] Nakano, M., Miyashita, O., Joti, Y., Suzuki, A., Mitomo, H., Niida, Y., Yang, Y., Yumoto, H., Koyama, T., Tono, K., Ohashi, H., Yabashi, M., Ishikawa, T., Bessho, Y., Ijiro, K., Nishino, Y. & Tama, F. (2022). *Optica*, **9**, 776–784.

[bb72] Neutze, R., Wouts, R., van der Spoel, D., Weckert, E. & Hajdu, J. (2000). *Nature*, **406**, 752–757. 10.1038/35021099 10963603

[bb73] Nishino, Y., Eltsov, M., Joti, Y., Ito, K., Takata, H., Takahashi, Y., Hihara, S., Frangakis, A. S., Imamoto, N., Ishikawa, T. & Maeshima, K. (2012). *EMBO J.* **31**, 1644–1653. 10.1038/emboj.2012.35 PMC3321210 22343941

[bb75] Pfeifer, M. A., Williams, G. J., Vartanyants, I. A., Harder, R. & Robinson, I. K. (2006). *Nature*, **442**, 63–66. 10.1038/nature04867 16823449

[bb76] Pfeiffer, F. (2018). *Nat. Photon.* **12**, 9–17.

[bb77] Pham, M., Yin, P., Rana, A., Osher, S. & Miao, J. (2019). *Opt. Express*, **27**, 2792–2808. 10.1364/OE.27.002792 30732311

[bb78] Raimondi, P., Benabderrahmane, C., Berkvens, P., Biasci, J. C., Borowiec, P., Bouteille, J.-F., Brochard, T., Brookes, N. B., Carmignani, N., Carver, L. R., Chaize, J.-M., Chavanne, J., Checchia, S., Chushkin, Y., Cianciosi, F., Di Michiel, M., Dimper, R., D’Elia, A., Einfeld, D., Ewald, F., Farvacque, L., Goirand, L., Hardy, L., Jacob, J., Jolly, L., Krisch, M., Le Bec, G., Leconte, I., Liuzzo, S. M., Maccarrone, C., Marchial, T., Martin, D., Mezouar, M., Nevo, C., Perron, T., Plouviez, E., Reichert, H., Renaud, P., Revol, J.-L., Roche, B., Scheidt, K.-B., Serriere, V., Sette, F., Susini, J., Torino, L., Versteegen, R., White, S. & Zontone, F. (2023). *Commun. Phys.* **6**, 82. 10.1038/s42005-023-01195-z PMC10124696 37124119

[bb79] Robinson, I. & Harder, R. (2009). *Nat. Mater.* **8**, 291–298. 10.1038/nmat2400 19308088

[bb80] Robinson, I. K., Vartanyants, I. A., Williams, G. J., Pfeifer, M. A. & Pitney, J. A. (2001). *Phys. Rev. Lett.* **87**, 195505. 10.1103/PhysRevLett.87.195505 11690423

[bb81] Rodenburg, J. M., Hurst, A. C., Cullis, A. G., Dobson, B. R., Pfeiffer, F., Bunk, O., David, C., Jefimovs, K. & Johnson, I. (2007). *Phys. Rev. Lett.* **98**, 034801. 10.1103/PhysRevLett.98.034801 17358687

[bb82] Rodriguez, J. A., Xu, R., Chen, C.-C., Huang, Z., Jiang, H., Chen, A. L., Raines, K. S., Pryor, A. Jr, Nam, D., Wiegart, L., Song, C., Madsen, A., Chushkin, Y., Zontone, F., Bradley, P. J. & Miao, J. (2015). *IUCrJ*, **2**, 575–583. 10.1107/S205225251501235X PMC4547825 26306199

[bb83] Rose, M., Bobkov, S., Ayyer, K., Kurta, R. P., Dzhigaev, D., Kim, Y. Y., Morgan, A. J., Yoon, C. H., Westphal, D., Bielecki, J., Sellberg, J. A., Williams, G., Maia, F. R. N. C., Yefanov, O. M., Ilyin, V., Mancuso, A. P., Chapman, H. N., Hogue, B. G., Aquila, A., Barty, A. & Vartanyants, I. A. (2018). *IUCrJ*, **5**, 727–736. 10.1107/S205225251801120X PMC6211532 30443357

[bb84] Sanzaro, S., Zontone, F., Grosso, D., Bottein, T., Neri, F., Smecca, E., Mannino, G., Bongiorno, C., Spinella, C., La Magna, A. & Alberti, A. (2019). *Nanomaterials*, **9**, 1300. 10.3390/nano9091300 PMC6781015 31514348

[bb85] Sayre, D. (1952). *Acta Cryst.* **5**, 843–843.

[bb86] Schropp, A., Hoppe, R., Patommel, J., Samberg, D., Seiboth, F., Stephan, S., Wellenreuther, G., Falkenberg, G. & Schroer, C. G. (2012). *Appl. Phys. Lett.* **100**, 253112.

[bb87] Shang, M., Liao, M., Li, Y., Lu, D., Deng, D., Zhang, C., Chen, H. & Lu, H. (2025). *Opt. Laser Technol.* **181**, 111726.

[bb88] Skjønsfjell, E. T. B., Chushkin, Y., Zontone, F. & Breiby, D. W. (2018*a*). *J. Synchrotron Rad.* **25**, 1162–1171. 10.1107/S160057751800588X 29979178

[bb89] Skjønsfjell, E. T. B., Kleiven, D., Patil, N., Chushkin, Y., Zontone, F., Gibaud, A. & Breiby, D. W. (2018*b*). *J. Opt. Soc. Am. A*, **35**, A7–A17. 10.1364/JOSAA.35.0000A7 29328079

[bb90] Soltau, J., Vassholz, M., Osterhoff, M. & Salditt, T. (2021). *Optica*, **8**, 818–823.

[bb91] Song, C., Ramunno-Johnson, D., Nishino, Y., Kohmura, Y., Ishikawa, T., Chen, C.-C., Lee, T.-K. & Miao, J. (2007). *Phys. Rev. B*, **75**, 012102.

[bb92] Stockmar, M., Cloetens, P., Zanette, I., Enders, B., Dierolf, M., Pfeiffer, F. & Thibault, P. (2013). *Sci. Rep.* **3**, 1927. 10.1038/srep01927 PMC3668322 23722622

[bb93] Stroke, G. & Falconer, D. (1964). *Phys. Lett.* **13**, 306–309.

[bb94] Sun, Y. & Singer, A. (2024). *Chem. Phys. Rev.* **5**, 031310.

[bb95] Takahashi, Y., Abe, M., Uematsu, H., Takazawa, S., Sasaki, Y., Ishiguro, N., Ozaki, K., Honjo, Y., Nishino, H., Kobayashi, K., Hiraki, T. N., Joti, Y. & Hatsui, T. (2023). *J. Synchrotron Rad.* **30**, 989–994. 10.1107/S1600577523004897 PMC10481278 37526992

[bb96] Takahashi, Y., Nishino, Y., Tsutsumi, R., Zettsu, N., Matsubara, E., Yamauchi, K. & Ishikawa, T. (2010). *Phys. Rev. B*, **82**, 214102. 10.1021/nl100891n 20402526

[bb97] Takazawa, S., Dao, D.-A., Abe, M., Uematsu, H., Ishiguro, N., Hoshino, T., Dam, H. C. & Takahashi, Y. (2023). *Phys. Rev. Res.* **5**, L042019.

[bb98] Thibault, P., Dierolf, M., Menzel, A., Bunk, O., David, C. & Pfeiffer, F. (2008). *Science*, **321**, 379–382. 10.1126/science.1158573 18635796

[bb99] Thibault, P., Elser, V., Jacobsen, C., Shapiro, D. & Sayre, D. (2006). *Acta Cryst.* A**62**, 248–261. 10.1107/S0108767306016515 16788265

[bb100] Tsai, E. H. R., Usov, I., Diaz, A., Menzel, A. & Guizar-Sicairos, M. (2016). *Opt. Express*, **24**, 29089–29108. 10.1364/OE.24.029089 27958573

[bb101] Ukleev, V., Yamasaki, Y., Morikawa, D., Kanazawa, N., Okamura, Y., Nakao, H., Tokura, Y. & Arima, T. (2018). *QuBS*, **2**, 3.

[bb102] Verezhak, M. (2016). PhD thesis, Université Grenoble Alpes, France.

[bb103] Vila-Comamala, J., Diaz, A., Guizar-Sicairos, M., Mantion, A., Kewish, C. M., Menzel, A., Bunk, O. & David, C. (2011). *Opt. Express*, **19**, 21333–21344. 10.1364/OE.19.021333 22108984

[bb104] Wilke, R. N., Vassholz, M. & Salditt, T. (2013). *Acta Cryst.* A**69**, 490–497.

[bb105] Wu, L., Juhas, P., Yoo, S. & Robinson, I. (2021). *IUCrJ*, **8**, 12–21. 10.1107/S2052252520013780 PMC7792998 33520239

[bb106] Yokoyama, Y., Yamasaki, Y., Okada, M. & Mizumaki, M. (2022). *J. Phys. Soc. Jpn*, **91**, 034701.

[bb107] Yoreo, J. J. De, Gilbert, P. U. P. A., Sommerdijk, N. A. J. M., Penn, R. L., Whitelam, S., Joester, D., Zhang, H., Rimer, J. D., Navrotsky, A., Banfield, J. F., Wallace, A. F., Michel, F. M., Meldrum, F. C., Cölfen, H. & Dove, P. M. (2015). *Science*, **349**, aaa6760. 10.1126/science.aaa6760 26228157

